# The Use of Integra Dermal Regeneration Template Versus Flaps for Reconstruction of Full-Thickness Scalp Defects Involving the Calvaria: A Cost–Benefit Analysis

**DOI:** 10.1007/s00266-016-0703-0

**Published:** 2016-10-03

**Authors:** M. Schiavon, M. Francescon, D. Drigo, G. Salloum, R. Baraziol, J. Tesei, E. Fraccalanza, F. Barbone

**Affiliations:** 1 Struttura Operativa Complessa di Chirurgia Plastica e Centro Ustioni, Azienda Ospedaliero-Universitaria S. Maria della Misericordia, Udine, Italy; 2 Istituto di Igiene ed Epidemiologia Clinica, Azienda Ospedaliero-Universitaria S. Maria della Misericordia, Udine, Italy

**Keywords:** Dermal Matrix, Integra, Cost benefit, Scalp reconstruction

## Abstract

**Background:**

INTEGRA^®^ Dermal Regeneration Template is a well-known and widely used acellular dermal matrix. Although it helps to solve many challenging problems in reconstructive surgery, the product cost may make it an expensive alternative compared to other reconstruction procedures. This retrospective study aims at comparing INTEGRA-based treatment to flap surgery in terms of cost and benefit.

**Patients and Methods:**

We considered only patients treated for scalp defects with bone exposure in order to obtain two groups as homogeneous as possible. We identified two groups of patients: 17 patients treated with INTEGRA and 18 patients treated with flaps. All patients were admitted in our institution between 2004 and 2010, and presented a defect of the scalp following trauma or surgery for cancer, causing a loss of the soft tissues of the scalp with bone exposure without pericranium. To calculate the cost in constant euros of each treatment, three parameters were evaluated for each patient: cost of the surgical procedure (number of doctors and nurses involved, surgery duration, anesthesia, material used for surgery), hospitalization cost (hospitalization duration, dressings, drugs, topical agents), and outpatient cost (number of dressing changes, personnel cost, dressings type, anti-infective agents). The statistical test used in this study was the Wilcoxon Mann–Whitney (*α* = 0.05).

**Results:**

No significant difference was characterized between the two groups for gender, age, presence of diabetes, mean defect size, and number of surgical procedures. All patients healed with good quality and durable closure. The median total cost per patient was €11,121 (interquartile range (IQR) 8327–15,571) for the INTEGRA group and €7259 (IQR 1852–24,443) for the flap group (*p* = 0.34). A subgroup of patients (six patients in the INTEGRA group and five patients in the flap group) showing defects larger than 100 cm^2^ were considered in a second analysis. Median total cost was €11,825 (IQR 10,695–15,751) for the INTEGRA group and €23,244 (IQR 17,348–26,942) for the flap group.

**Conclusion:**

Both treatments led to a good healing of the lesions with formation of soft and resistant tissue. No significant difference was characterized between the two groups for days of hospitalization and costs. In cases of patients with defects larger than 100 cm^2^ for whom major surgery is needed, the treatment with INTEGRA seemed to be less expensive than the treatment with free flaps or pedicle flaps.

**Level of Evidence V:**

This journal requires that authors assign a level of evidence to each article. For a full description of these Evidence-Based Medicine ratings, please refer to the Table of Contents or the A5 online Instructions to Authors.www.springer.com/00266.

## Introduction

INTEGRA^®^ Dermal Regeneration Template has been commercialized since the 1980s. Its use was initially described by Burke and colleagues in 1981 [1], and it is now an important tool for the treatment of burns and scar contractures [2, 3]. INTEGRA is also used for the reconstruction of soft tissue loss following trauma, cancer removal, and scar revision of all anatomical sites [4–6]. The specificity of INTEGRA is that it is perfectly integrated where it is placed, regenerating a tridimensional structure, known as neo-dermis, in which fibroblasts, lymphocytes, macrophages, and neovascularization are clearly detectable [3, 7–9]. This type of dermal regeneration template offers multiple advantages: it allows immediate closure of the wound, thus avoiding fluid loss and restoring the functional barrier of the skin. It also prepares the wound before the positioning of a split-thickness skin graft. It is applicable in anatomical regions in which a graft placement alone would not be preferred, such as on bone- and tendon-exposed areas [4, 10], and improves the final outcome and feature of the scar [11–13]. The positioning of the matrix is relatively simple with a reduction of operating time. It allows the reduction of hospitalization time and of surgical sequelae for the patient [6, 14]. However, INTEGRA could also be susceptible to infection, needs a second procedure for the coverage of the matrix with a skin graft, and has a relatively high cost compared to autografts. The latter is probably the main reason for its limited use in the clinical practice: in fact, not all hospital administrations are willing to authorize its purchase.

From the clinical literature perspective, only one cost comparison study about INTEGRA use was identified. This study compared the use of INTEGRA to split-thickness skin grafts for the treatment of chronic wounds [15]. No significant difference was characterized between the groups in terms of charges, time to healing, narcotic use, or antibiotic use, thus showing that the use of INTEGRA could be an economically valid alternative treatment for chronic wounds. In this context, it seems very interesting to perform similar studies with larger samples and in other indications.

This retrospective study was set up considering patients affected by diseases for which INTEGRA is usually used in a Department of Plastic Surgery such as tumor removal, consequences of traumatic lesions, or burns. The patients considered for this cost analysis study underwent surgery for the reconstruction of scalp defects. Scalp reconstruction was chosen because this anatomical area presents a good vascular supply which is not influenced by alterations caused by vascular disease or habits, and rehabilitation does not require the patient to stay in bed. Total costs for the management of these patients, from preparation of the wound bed to complete wound healing, were calculated and compared between patients who were treated with INTEGRA and patients who had various forms of flap surgery.

## Patients and Methods

The study was a retrospective study. All consecutive patients (38 patients) with neoplastic or traumatic lesions were identified who underwent scalp reconstructive surgery at our operative unit between 2004 and 2010. Among them, 18 patients had received INTEGRA^®^ Dermal Regeneration Template bilayer followed by a secondary split-thickness skin graft, and 20 patients had undergone flap surgery (either with local flaps or microsurgical flaps or tissue expansion) for the coverage of the tissue loss. All patients presented with some area of denuded calvarian bone without periosteum. The choice of the reconstruction technique (INTEGRA or flap reconstruction) was related to the general condition of the patients: i.e., some patients not eligible for major surgical procedures or some patients with bad tissue conditions (presence of scars or irradiated tissues) were treated with INTEGRA. The dermal matrix was placed on the wound during the first surgery, trimmed to the size of the defect, and stapled. We milled the bone until we had some bleeding before covering the wound bed with INTEGRA.

No vacuum therapy was performed for the study patients because the scalp is a very well vascularized structure. Among the 20 patients who underwent flap reconstruction, there were 14 pedicle flaps, 3 free flaps (latissimus dorsi), and 1 skin expansion procedure (2 patients with too small area treated were excluded). Patients of the INTEGRA group were discharged quite rapidly after the first surgery when allowed by their general condition. Following the secondary split-thickness skin graft procedure, patients were discharged after the first dressing change. After each procedure, the first dressing change was performed on day 5. Subsequent dressing changes were performed as frequently as needed. All dressing changes were performed either in the hospital or in the author’s practice depending on when the patient was discharged. Patients of the flap group were discharged depending on the type of surgery performed and their general condition. Outpatients were discharged on the day of surgery and came back to the author’s practice for the first dressing change 3–5 days after the operation. Inpatients usually had their first dressing change 2–3 days after surgery.

For each patient, personal data, date and duration of hospitalization, and comorbidities were gathered. Concerning the lesions, if tumors were removed, the dimensions of the lesions were recorded as well as the size of tissue loss for both neoplastic and traumatic lesions. As for the surgery, besides the type of surgery, the overall operating time, the number of surgeons involved, the type of anesthesia administered, and the materials used were also recorded, as well as the medications and the supplies used for the treatment during hospitalization and after discharge of the patient, until complete wound healing.

To achieve an overall cost calculation in constant euros, the management software used by the warehouse of the hospital (Ascot Web, Insiel S.p.a., Trieste, Italy) provided the unit costs of the materials and drugs. The hospital management also provided all the data related to the hourly hospital staff and operating room costs. The surgical procedures considered for the cost analysis were in all cases removal of the lesion, plus one of the following procedures: subsequent INTEGRA placement and coverage with skin graft in a second stage, or repair with scalp flap, or delayed repair with pedicle flap after skin expansion or not, or repair with free flap.

Descriptive analysis was performed, and median, interquartile range (IQR), mean, and standard deviation (SD) were calculated. The cost and duration of hospitalization (inpatient and outpatient hospitalization) in each group were compared by the Wilcoxon–Mann–Whitney test. The alpha level was set to 0.05 for all tests. The statistical analysis was performed using SAS 9.2 (SAS Institute Inc., Cary, North Carolina, USA).

## Results

Among the patients selected for inclusion in this study, one patient treated with INTEGRA died of a stroke 2 weeks after the surgery and was not kept for the analysis. To increase the homogeneity of the group, it was also decided to exclude two patients of the group treated with flaps because the size of their lesions was too small (inferior to 3 cm^2^) to be compared with the other patients. The present sample compiles data from 17 patients treated with INTEGRA (8 men and 9 women) and 18 treated with flaps (9 men and 9 women).

The median age of the patients included in the evaluation at the time of their first surgery was 73 years (IQR 48–77) in the INTEGRA group and 71 years (IQR 56–84) in the flap group. Diabetes was reported for three patients (one patient in the INTEGRA group and two in the flap group). Operated lesions were mainly neoplastic (76.5 % of the cases in the INTEGRA group and 72.2 % in the flap group). The median size of the defect was 56 cm^2^ (IQR 28–100) for the INTEGRA group and 20 cm^2^ (IQR 7–78) for the flap group. The number of procedures performed for each patient varies from 2 (76.5 % of the cases) to 3 (23.5 % of the cases) for the patients of the INTEGRA group and from 1 (77.8 % of the cases) to 4 (5.6 % of the cases) for the patients treated with flaps (Table 1).

**Table 1:** Description of patients and procedures

		INTEGRA (*n* = 17)	Flap (*n* = 18)	Total (*n* = 35)	*p* value
Age	Median (IQR)	73 (48–77)	71 (56–84)	71 (48–84)	0.9
Gender	Male (%)	8 (47.1)	9 (50)	17 (48.6)	–
	Female (%)	9 (52.9)	9 (50)	18 (51.4)	
Diabetes	(%)	1 (5.9)	2 (11.1)		–
Type of lesion	Neoplastic (%)	13 (76.5)	13 (72.2)	26 (74.3)	–
	Traumatic (%)	3 (17.7)	–	3 (8.6)	
	Degenerative (%)	–	1 (5.6)	1 (2.9)	
	Iatrogenic (%)	–	4 (22.2)	4 (11.4)	
	Infection (%)	1 (5.9)	–	1 (2.9)	
Lesion dimension in cm^2^	Median (IQR)	28 (10.0–78.5)	10.8 (3.0–35.0)	–	0.22
Defect dimension in cm^2^ (after debridement)	Median (IQR)	56 (28.0–100.0)	20 (7.0–78.5)	–	0.22
Number of procedures (%)	1	0	14 (77.8)	14 (40.0)	–
	2	13 (76.5)	2 (11.1)	15 (42.9)	
	3	4 (23.5)	1 (5.6)	5 (14.3)	
	4	0	1 (5.6)	1 (2.9)	

A total of 56 inpatient admissions were recorded: 35 for patients of the INTEGRA group and 21 for patients of the flap group. Patients of the INTEGRA group were all hospitalized twice: once for matrix implantation and once for split-thickness skin grafting. The median value for the total duration of inpatient hospitalization was 6.5 days (IQR 5.0–9.5) for the INTEGRA group and 14.0 days (IQR 10.0–28.0) for the flap group. Some outpatient admissions were also reported. The median duration of the outpatient follow-up was 10.5 days (IQR 10.0–11.0) for the INTEGRA group and 6.5 days (IQR 4.0–8.0) for the flap group (i.e., for one patient, a day hospital follow-up of 10 days corresponded to 10-day hospital admissions during the treatment). Hospitalization durations are presented in Table 2. The median surgery duration was 40.0 min (IQR 10.0–55.0) for the INTEGRA group and 70.0 min (IQR 10–170.0) for the flap group (Table 2). The long surgery durations encountered in the flap group were related to difficult tumor excision and subsequent flap reconstruction.

**Table 2:** Hospitalization durations calculated for each individual admission (one patient could have more than one admission)

	INTEGRA (*n* = 17)		Flap (*n* = 18)	
	Mean (SD)	Median (IQR)	Mean (SD)	Median (IQR)
Inpatient hospitalization duration (days)	9.4 (8.6)	6.5 (5.0–9.5)	21.8 (19.9)	14.0 (10.0–28.0)
Outpatient hospitalization duration (days)	10.5 (0.7)	10.5 (10.0–11.0)	6.0 (2.4)	6.5 (4.0–8.0)
Surgery duration (min)	48.2 (32.0)	40.0 (10.0–55.0)	139.2 (167.7)	70.0 (10.0–170.0)

The number of inpatient admissions considered was 35 for the INTEGRA group and 21 for the flap group. The number of outpatient admissions considered was three for the INTEGRA group and four for the flap group

Since the treatment with INTEGRA requires two surgical procedures (first, matrix positioning and second, coverage with split-thickness skin graft 1 month later), a second analysis was performed pulling inpatient hospitalizations as one. In that case, the median duration of hospitalization reached 15.0 days for the INTEGRA group (IQR 12.0–23.5) and 15.0 days (IQR 6.0–41.0) for the flap group. No significant difference was evidenced between the two groups (*p* value = 0.93).

**Fig. 1:**
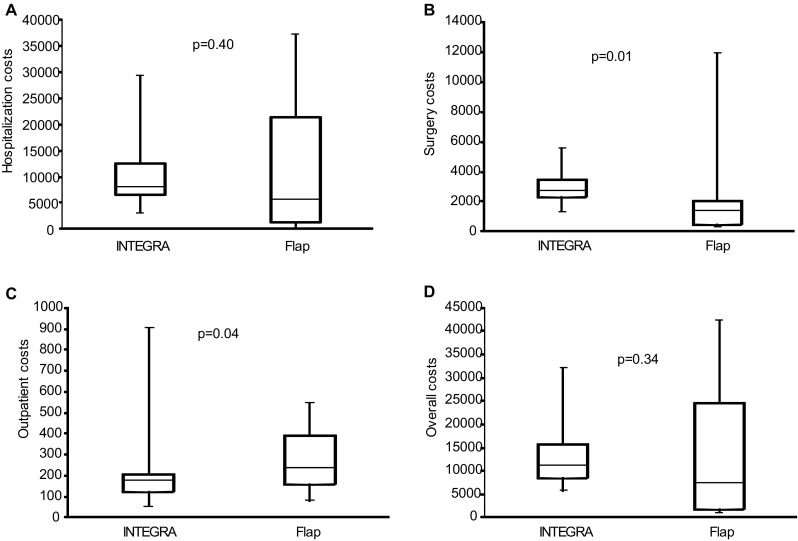
Costs in euros presented per group for hospitalization (**a**), surgery (**b**), outpatients (**c**), and overall costs (**d**). Results are presented as median, IQR, and minimal and maximal values. Wilcoxon–Mann–Whitney test were performed

The overall costs of hospitalization (in- and outpatient), outpatient management, and surgery are presented in Fig. 1. The median overall cost was 11,121.2€ (IQR 8326.9–15,751.1) for patients of the INTEGRA group and 7259.7€ (IQR 1852.1–24,443.4) for patients of the flap group. No significant difference was characterized between the two groups (*p* = 0.33).

When considering only patients who presented with a lesion larger than 100 cm^2^ (six in the INTEGRA group and five in the flap group), the median defect size was 120.0 cm^2^ (IQR 106.5–145.5) for patients of the INTEGRA group and 154.0 cm^2^ (IQR 113.0–154.0) for patients of the flap group. The median overall cost was 11,824.99€ (IQR 10,694.93–15,751.12) for patients of the INTEGRA group and 23,244.47€ (IQR 17,348.38–26,942.29) for patients of the flap group (Table 3). For these patients who usually need to be treated with microsurgery reconstructive procedures because of the size of the defect, overall costs seem to be twice as high for patients of the flap group compared to patients of the INTEGRA group.

**Table 3:** Costs in euros for the subgroup of patients with larger size lesions (above 100 cm^2^)

	INTEGRA (*n* = 6)		Flap (*n* = 5)	
Type of cost	Mean (DS)	Median (IQR)	Mean (DS)	Median (IQR)
Hospital stay	9572.5 (3030.8)	8205.0 (7658.0–13,128.0)	17,066.4 (9939.8)	19,692.0 (8205.0–21,333.0)
Surgery costs	3066.2 (583.4)	2880.1 (2693.9–3418.6)	6305.0 (4174.5)	5609.3 (3113.4–9143.4)
Outpatient costs	287.8 (305.8)	184.3 (124.1–222.3)	353.4 (108.4)	389.62 (231.6–439.1)
Total costs	12,926.5 (3210.9)	11,825.0 (10,694.9–15,751.1)	23,583.5 (12,675.0)	23,244.5 (17,348.4–26,942.3)

Outpatient and inpatient admissions were pooled for hospital stay cost calculations

## Discussion

The reconstruction of scalp defects requires an immediate coverage of the skull to preserve the anatomical features and consistency of the surrounding tissues as much as possible. These procedures can be particularly challenging in the presence of large defects, in the case of cranial bone exposure, and in the presence of scar tissues due to previous surgeries or after radiotherapy. There are different publications showing the efficacy of INTEGRA in providing an adequate reconstruction alternative for challenging cases compared to traditional reconstructive procedures [4, 16, 17].

The aim of the present retrospective study was to compare the cost of two main surgical alternatives in scalp reconstruction procedures where bone denuded of periosteum was exposed. Data about surgery, hospitalization, and management were gathered in patients treated either with INTEGRA or with flaps. No significant difference was characterized between the two groups of patients concerning their age, the reason, and the size of the scalp defect.

From the analysis of the duration of hospitalization, it was observed that admissions for the patients treated with INTEGRA seemed shorter but, at the same time, patients were admitted in the hospital at least twice because of the 2-step procedure. Overall, no significant difference was characterized between the two groups for the duration of hospitalization. The durations of hospitalization reported in this study could be considered high compared to the literature [4, 18] where durations range from 2 to 7 days on average. However, patients of the study were usually not discharged because of their general condition and not because of complications related to skin graft procedures.

Costs in constant euros were compared between the two groups for hospital stay, surgery, and outpatient management. Outpatient management cost was calculated considering material and human resources costs. While no significant difference was found between the two groups for hospital stay cost (*p* = 0.40), surgery cost was significantly higher for the INTEGRA group (*p* = 0.01) and outpatient cost significantly higher for the flap group (*p* = 0.04). However, even with a higher median lesion size in the INTEGRA group, no significant difference was characterized between the two groups for the overall cost (*p* = 0.34).

One limitation of this study is the relatively low number of patients included in the analysis. Also, the size of the lesions included in the analysis varied greatly from a few cm^2^ to large defects of more than 100 cm^2^. As a consequence, the overall cost calculated from patients’ data is also relatively variable. In this context, it was very interesting to analyze separately the more severe patients with defects larger than 100 cm^2^. Despite not having a statistical test that could be carried out because of the low number of patients concerned, the use of INTEGRA seemed to reduce the overall cost of the reconstruction compared to the use of flaps. This preliminary result which should be confirmed in a larger sample of patients somehow confirms a study published in 2012 [19] which concluded that INTEGRA was a reasonable tissue-engineered alternative to free tissue transfer in medically compromised patients with complex lower extremity wounds (denuded tendon and bone exposure). However, in this paper, INTEGRA and Negative Pressure Therapy (NPT) were associated with reduced problems due to poor vascularization in the legs. In our study, we did not use NPT because we treated wounds of the scalp, which have a good blood supply.

Today, in some cases, reconstruction with INTEGRA can be performed in one step using INTEGRA Single Layer, and this change will allow in the future a different look at the costs for the INTEGRA procedure.

Another limitation of the results is related to the single center design of the study. Also, cost calculation was based on our hospital practices, and it is difficult to know how reproducible it would be in another hospital or another country. The retrospective design of the study could have led to an information bias. Thus, the results presented here have to be confirmed in larger scale prospective studies and also in other countries.

Importantly, although this was not completely evaluated in this study, the procedure with INTEGRA probably limits discomfort for the patient because it allows for decreased surgical time, with reduced donor site morbidity (only split-thickness skin graft is required) and fast recovery rate. This aspect is particularly important for elderly patients or for patients with comorbidities who would have to face serious risks if treated with major reconstructive surgical procedures requiring microsurgery.

**Fig. 2:**
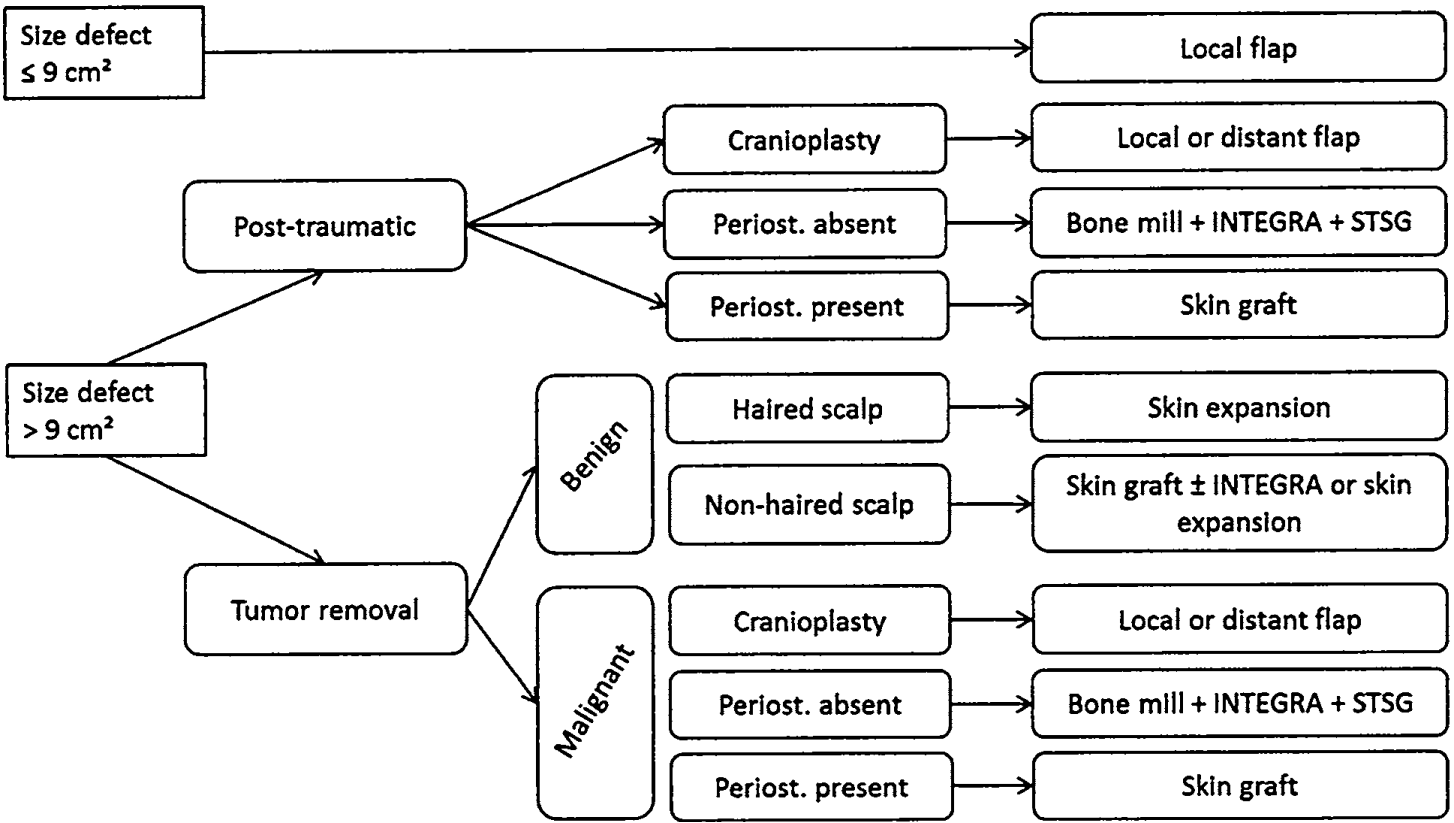
Algorithm for surgical treatment of scalp defects (STSG is for “split-thickness skin graft”)

Each therapeutic choice during the treatment of a patient has to be made considering patient-related factors like age, comorbidities, and logistic factors (such as problems in coming to the office for dressing changes). Since we introduced the use of INTEGRA in our armamentarium for scalp reconstruction, our algorithm for surgical treatment is as follows (Fig. 2). We perform local flaps for defects smaller than 3 × 3 cm and especially if hair is present. We also use flaps to cover prosthetic material for example in case of cranioplasty. In young people with non-malignant pathologies and haired scalp, we prefer skin expansion before lesion removal. In case of elderly people with important comorbidities and large defects, we prefer using INTEGRA and split-thickness skin graft, because it decreases the duration of the surgical procedure and anesthesia.

## Conclusion

No significant difference was characterized for hospitalization duration and costs between the use of INTEGRA Dermal Regeneration Template and the use of flaps for reconstruction of scalp defects. Moreover, it seems that in patients with larger defects requiring challenging surgical procedures, the use of INTEGRA decreases the overall cost of treatment by a factor of two. The common feeling that INTEGRA may be an expensive treatment is not confirmed by our cost analysis when considering the total cost of the procedure, hospitalization, and outpatient costs. INTEGRA appears to be a cost-effective therapeutic alternative for reconstruction of scalp defects compared to flap surgery. The results of this study need to be confirmed in a larger multicenter study.
